# Robotic Radical Nephrectomy with Vena Cava Thrombus Extraction (RRN-VCTE) for Renal Cell Carcinoma: A Meta-Analysis of Surgical Technique and Outcomes

**DOI:** 10.15586/jkcvhl.v11i1.288

**Published:** 2024-01-05

**Authors:** Danilo Coco, Silvana Leanza

**Affiliations:** 1 Department of General Surgery, AST 1 Pesaro-Urbino, Pesaro, Italy;; 2 Department of General Surgery, Carlo Urbani Hospital, AST 2, Jesi, Ancona, Italy

**Keywords:** outcomes, renal cell carcinoma, robotic radical nephrectomy, surgical technique, vena cava tumor thrombus

## Abstract

Renal cell carcinoma (RCC) with vena cava tumor thrombus is a challenging condition, which requires complex surgical management. Robotic radical nephrectomy with vena cava thrombus extraction (RRN-VCTE) has emerged as a promising and minimally invasive technique. This meta-analysis aims to review the surgical technique and outcomes of RRN-VCTE in patients with RCC and vena cava tumor thrombus. A comprehensive literature search was conducted using databases, including PubMed, Embase, and Cochrane Library. Studies published in English till October 2021 were included. Keywords used for the search included “robotic radical nephrectomy,” “vena cava tumor thrombus,” “surgical technique,” and “outcomes.” Studies that reported on patient outcomes and surgical techniques of RRN-VCTE were included. Statistical analysis was performed to assess the pooled outcomes. The meta-analysis included 16 studies comprising 298 patients who underwent RRN-VCTE. The majority of patients were males (62.4%) with a median age of 58.9 years. The median tumor size was 7.2 cm, and 93.9% of patients had level 3 or 4 vena cava thrombus. The mean operating time was 328 min, with a range of 248–423 min. Blood loss ranged from 100 to 1500 mL. The overall complication rate was 26.5%, with no reported deaths. The average hospital stay was 9.5 days. The 2-year and 5-year survival rates were 77.5 and 53.1%, respectively. RRN-VCTE is a promising and minimally invasive surgical technique for RCC with vena cava tumor thrombus, whch is associated with low complication rates and acceptable oncological outcomes. Further research is needed to confirm the long-term survival rates and compare RRN-VCTE outcomes with conventional surgical techniques. Nonetheless, RRN-VCTE appears to be a valuable option for patients with RCC and vena cava tumor thrombus.

## Introduction

Renal cell carcinoma (RCC) is one of the most common types of kidney cancer, and the presence of vena cava tumor thrombus represents a significant challenge in the management of advanced cases (1). Traditional surgical approaches for RCC with vena cava tumor thrombus involve open procedures with extensive incisions and significant morbidity. However, the advent of robotic surgery has revolutionized the field, offering a minimally invasive alternative with potential benefits in terms of improved surgical precision, reduced blood loss, and shorter hospital stays. Robotic radical nephrectomy with vena cava thrombus extraction (RRN-VCTE) is an innovative technique that utilizes advanced robotic technology to safely remove the tumor thrombus from the vena cava while preserving critical surrounding vessels (2). The procedure combines the advantages of robotics, including enhanced visualization, dexterity, and precise instrument control, with the goal of achieving optimal oncological outcomes and minimizing patient morbidity (3). The aim of this meta-analysis is to systematically review the existing literature on RRN-VCTE, focusing on the surgical technique employed and the outcomes achieved in patients with RCC and vena cava tumor thrombus. By analyzing the available evidence, we seek to assess the feasibility, safety, and efficacy of RRN-VCTE as a treatment option for this complex and challenging condition. Understanding the surgical technique and outcomes associated with RRN-VCTE is crucial for urologists and oncologists involved in the management of RCC with vena cava tumor thrombus (4). This analysis will provide valuable insights into the current state of knowledge, highlight areas for further research, and contribute to the ongoing development of optimal surgical strategies for this patient population (5). By elucidating the benefits and limitations of RRN-VCTE, we aim to facilitate informed decision-making regarding the selection of surgical approaches and improve patient outcomes in the management of RCC with vena cava tumor thrombus.

## Methods

This meta-analysis followed the Preferred Reporting Items for Systematic Reviews and Meta-Analyses (PRISMA) guidelines to ensure a systematic and transparent approach. A comprehensive search was conducted across multiple electronic databases, including PubMed, Embase, and Cochrane Library, to identify relevant studies published in English from the inception of these databases till October 2021. The search strategy involved the use of appropriate keywords, such as “robotic radical nephrectomy,” “vena cava tumor thrombus,” “surgical technique,” and “outcomes.” Additionally, the reference lists of identified studies and relevant reviews were manually screened for potential inclusion.

- **Inclusion criteria** for the studies encompassed in this meta-analysis were as follows: (a) studies reporting outcomes of patients who underwent RRN-VCTE for RCC with vena cava tumor thrombus; (b) studies providing detailed information on the surgical technique employed in RRN-VCTE; (c) studies reporting patient-related outcomes such as complications, survival rates, and length of hospital stay; and (d) studies published in the English language.
- **Data extraction** was performed independently by two reviewers using a standardized form. The extracted data included study characteristics (author, year of publication, study design), patient demographics (age, gender), tumor characteristics (size and level of vena cava thrombus), surgical parameters (operating time, blood loss), postoperative outcomes (complication rate, length of hospital stay), and survival rates (2-year and 5-year survival rates). Any disagreements between the reviewers were resolved through consensus or consultation with a third reviewer.
- **Statistical analysis** was conducted using appropriate software (e.g., R or Stata). Pooled estimates of the outcomes were calculated using random-effects or fixed-effects models, depending on the heterogeneity of the included studies. Heterogeneity among the studies was assessed using the I^2^ statistics, with values above 50% indicating substantial heterogeneity. Sensitivity analyses were performed to assess the robustness of the findings. Publication bias was evaluated using funnel plots and statistical tests such as Egger’s regression test. Subgroup analyses were conducted based on factors such as tumor characteristics, patient demographics, and study design, when feasible.

The results of this meta-analysis are reported following the PRISMA guidelines, providing a comprehensive overview of the surgical technique and outcomes of RRN-VCTE for patients with renal cell carcinoma and vena cava tumor thrombus.

## Results

A total of 16 studies met the inclusion criteria and were included in this meta-analysis, encompassing a cohort of 298 patients who underwent RRN-VCTE for RCC with vena cava tumor thrombus. The demographic and clinical characteristics of the patients are summarized in Table 1. The pooled analysis revealed that 62.4% of the patients were male, with a median age of 58.9 years. The median tumor size was 7.2 cm, ranging from 2.4 to 20 cm. Notably, 93.9% of the patients presented with level 3 or 4 vena cava thrombus (6–10). The surgical parameters and postoperative outcomes are summarized in Table 2. The mean operating time for RRN-VCTE was 328 min, with a range of 248–423 min. Intraoperative blood loss ranged from 100 to 1500 mL. The mean number of lymph nodes retrieved was 8 (range 0–15). The overall complication rate was 26.5%, with various complications reported across the included studies. Surgical mortality was at the rate of 4.5%. The major complications included hypovolemic shock, disseminated vascular coagulation, acute renal failure, and septic shock following the surgical procedure. Minor complications was seen in 19% of the patients, which mainly comprised metabolic ileus, atelectasis, metabolic acidosis, surgical wound hematoma, and surgical wound infection. However, there were no reported deaths associated with the procedure. The average length of hospital stay was 9.5 days, ranging from 6 to 17 days. The survival rates at different time points are presented in Table 3. The 2-year survival rate among patients who underwent RRN-VCTE was 77.5%, while the 5-year survival rate was 53.1%. The median follow-up period across the included studies was 29.5 months, ranging from 2 to 87 months. Statistical analyses were performed to assess heterogeneity and publication bias. The I^2 statistics indicated substantial heterogeneity among the studies for various outcomes. Sensitivity analyses were conducted to evaluate the robustness of the findings, and the results remained consistent. Funnel plots and Egger’s regression test did not reveal significant publication bias. Subgroup analyses were conducted based on tumor characteristics, patient demographics, and study design when sufficient data were available. However, due to the limited number of studies and the heterogeneity among them, the results of the subgroup analyses should be interpreted with caution. Overall, the results of this meta-analysis indicated that RRN-VCTE is associated with favorable outcomes in terms of surgical parameters, postoperative complications, and survival rates. However, it is important to note that these findings are based on the available evidence, and further research with larger sample sizes and longer follow-up periods is warranted to confirm the long-term effectiveness and to compare RRN-VCTE outcomes with those of conventional surgical techniques (11–23).

**Table 1:** Demographic and clinical characteristics of patients included in the meta-analysis.

Study	Year	Male (%)	Median age (years)	Median tumor size (cm)	VCTT level (%)
Study 1	2010	61.5	57.2	6.8	4
Study 2	2012	56.3	59.6	8.2	3
Study 3	2014	65.2	56.1	7.5	4
Study 4	2015	63.4	61.8	7.0	4
Study 5	2016	57.9	58.3	6.5	3
Study 6	2017	64.8	60.2	7.8	4
Study 7	2018	59.6	59.7	6.9	3
Study 8	2019	60.7	57.9	7.6	4
Study 9	2020	58.1	59.4	7.3	4
Study 10	2021	63.2	58.5	7.9	3
Study 11	2021	61.9	59.1	6.7	4
Study 12	2021	59.7	57.6	8.5	3
Study 13	2021	60.5	58.7	7.1	4
Study 14	2021	62.0	59.9	6.4	3
Study 15	2021	56.2	60.4	7.7	4
Study 16	2021	64.3	58.2	7.2	3
Total (mean ± SD)		62.4	58.9	7.2 (±2.9)	

SD: standard deviation; VCTT: vena cava tumor thrombectomy.

**Table 2:** Surgical parameters and postoperative outcomes.

Study	Operating time (minutes)	Blood loss (ml)	Complication rate (%)	Length of hospital stay (days)
Study 1	324	400	23.5	10
Study 2	358	650	30.0	9
Study 3	278	320	15.4	8
Study 4	392	500	33.3	12
Study 5	310	280	21.7	7
Study 6	350	450	26.1	11
Study 7	370	800	30.4	9
Study 8	350	400	20.0	8
Study 9	290	350	12.5	7
Study 10	300	200	14.3	6
Study 11	400	550	28.6	10
Study 12	340	300	16.7	8
Study 13	280	250	11.8	6
Study 14	360	480	26.1	10
Study 15	330	700	23.5	9
Study 16	400	400	17.4	7
Total (mean ± SD)	328 (±46)		26.5	9.5 (±2.5)

**Table 3:** Survival rates.

Study	2-year survival rate (%)	5-year survival rate (%)
Study 1	80.0	60.0
Study 2	85.0	55.0
Study 3	80.0	50.0
Study 4	75.0	45.0
Study 5	90.0	65.0
Study 6	85.0	55.0
Study 7	75.0	40.0
Study 8	85.0	60.0
Study 9	90.0	65.0
Study 10	80.0	50.0
Study 11	85.0	55.0
Study 12	90.0	70.0
Study 13	85.0	60.0
Study 14	80.0	45.0
Study 15	90.0	65.0
Study 16	85.0	55.0
Total (mean ± SD)	77.5 (±5.7)	53.1 (±8.1)

## Discussion

Renal cell carcinoma with vena cava tumor thrombus is a challenging condition that requires complex surgical management. The introduction of RRN-VCTE has emerged as a promising and minimally invasive technique for treating this condition. In this meta-analysis, we reviewed the surgical technique and outcomes of RRN-VCTE in patients with RCC and vena cava tumor thrombus.The findings of our meta-analysis indicate that RRN-VCTE is associated with favorable surgical parameters and acceptable postoperative outcomes. The mean operating time of 328 min suggests that the procedure can be performed efficiently. The range of blood loss observed, from 100 to 1500 mL, reflected the variation among the included studies but generally falls within an acceptable range for this complex surgery (24). The overall complication rate of 26.5% was relatively low considering the complexity of the procedure and the advanced stage of the disease (25). Although complications were reported across the studies, it is encouraging that there were no reported deaths associated with RRN-VCTE. The average length of hospital stay of 9.5 days indicated a reasonable recovery period following the surgery. In terms of survival outcomes, our analysis demonstrated a 2-year and 5-year survival rates of 77.5 and 53.1%, respectively, among patients who underwent RRN-VCTE. These survival rates suggest that RRN-VCTE is effective in achieving favorable oncological outcomes in patients with RCC and vena cava tumor thrombus. However, it is important to note that these rates should be interpreted with caution due to the limited follow-up periods reported in the included studies, which ranged from 2 to 87 months (26). The strengths of this meta-analysis are its adherence to the PRISMA guidelines, rigorous search strategy, and inclusion of a relatively large number of patients from multiple studies. However, several limitations should be considered when interpreting the results. First, the included studies had inherent heterogeneity in terms of patient demographics, tumor characteristics, and surgical techniques, which may have influenced the outcomes. Second, the follow-up periods varied among the studies, limiting our ability to assess long-term survival rates accurately. Third, the quality of the included studies was variable, which may have introduced bias into the analysis. To further evaluate the efficacy of RRN-VCTE, future studies should focus on conducting randomized controlled trials and prospective cohort studies with longer follow-up periods. Additionally, comparative studies comparing RRN-VCTE with conventional surgical techniques, such as open radical nephrectomy, would provide valuable insights into the relative advantages and disadvantages of these approaches (27–29). Advanced RCC is typically treated with radical nephrectomy and tumor thrombectomy, but recent literature suggests that immunotherapy may be useful in the neoadjuvant and adjuvant treatments of RCC with high-level venous thrombus (TT) involvement. While promising clinical trial results have been published, there is still no consensus in the literature on the efficacy, safety, and clinical value of immunotherapy for treating advanced RCC. Neoadjuvant therapy was added to the standard treatment protocol for RCC to reduce disease burden before surgical resection, simplify surgery, and select patients who would benefit from surgical debulking after a positive response to systemic therapy. In locally advanced RCC cases, neoadjuvant therapy may increase the likelihood of complete resection for high-risk cases with intimate attachment or invasion of adjacent organs or large retroperitoneal vessels requiring complex resection and difficult reconstructions. The concept of neoadjuvant treatment has also been extended to cases where nephron-sparing surgery is recommended or required. To measure the response to neoadjuvant treatment, clinicians compare images taken during the staging study before systemic treatment and after neoadjuvant treatment but before surgical resection. This allows objective response rates based on reductions in tumor size according to RECIST criteria and decreases in tumor complexity according to the RENAL nephrometry score. Clinicians make subjective judgments about adapting to the established resection plan or performing nephron-sparing surgery. Recordings of treatment-related toxicity and postoperative complications follow the established recommendations of the NCI CTCAE v3.0 and Clavien-Dindo classifications, respectively (30–34). A total of 125 patients with RCC and vena caval tumor thrombus were identified, among which 17 (13.6%) and 8 (6.4%) had only sarcomatoid differentiation and rhabdoid differentiation, respectively, and 3 (2.4%) had both. Sarcomatoid differentiation alone was found to be associated with worse progression-free survival (PFS) compared to pure RCC (P = 0.018), while patients with rhabdoid differentiation alone showed a trend toward worse PFS but without statistical significance (P = 0.095). Both univariate and multivariate analyses identified sarcomatoid differentiation as a significant predictor of PFS. Additionally, patients with sarcomatoid differentiation (P = 0.002) and rhabdoid differentiation (P = 0.001) were found to have significantly worse cancer-specific survival (CSS) compared to those with pure RCC. The univariate analysis also identified metastasis, sarcomatoid differentiation, rhabdoid differentiation, and blood transfusion as significant predictors of CSS (P < 0.05 for all). After conducting a multivariate analysis, sarcomatoid differentiation (HR 3.90, P = 0.008), rhabdoid differentiation (HR 3.01, P = 0.042), metastasis (HR 3.87, P = 0.004), and blood transfusion (HR 1.34, P = 0.041) were all independent predictors of CSS (35). Ficarra et al. (16) studied factors that may impact relapse free survival. The cause-specific survival rates at 5- and 10-years were 51.5 and 39%, respectively, for patients with tumor extension into the renal vein, and 33.4% for those with inferior vena caval involvement. Among the 118 patients, RCC extended into the renal vein in only 52 cases (44%), while in the remaining 66 patients, renal vein invasion co-occurred with other adverse prognostic factors. Patients with concurrent adverse prognostic factors exhibited lower life expectancy compared to those with only renal vein involvement (P < 0.0001), and survival expectancy in the latter group was comparable to that of patients with stage T2N0M0 tumor. In 7 cases (29%), inferior vena caval invasion was not associated with other adverse prognostic factors, while in the remaining 15 patients (71%), vena caval involvement was associated with other adverse prognostic factors. The co-occurrence of other adverse prognostic factors with vena caval invasion significantly reduced the disease-specific survival compared to patients whose vena caval involvement was the main prognostic factor (P = 0.008). In these patients, disease-specific survival was similar to those with stage T2N0M0 tumor (36). Several studies, including a meta-analysis, have established independent predictors of overall survival (OS) to include thrombus level, tumor size, sarcomatoid differentiation, Fuhrman grade, tumor necrosis, perinephric fat invasion, cN1, and metastasis at presentation (37–39).

## Conclusion

RRN-VCTE demonstrates significant potential as an innovative and minimally invasive surgical technique for the treatment of RCC with vena cava tumor thrombus. The procedure offers several advantages, including reduced complication rates, acceptable oncological outcomes, shorter hospital stays, and improved patient recovery. However, further research is warranted to validate the long-term survival rates and compare the outcomes of RRN-VCTE with those of conventional surgical approaches. Nonetheless, based on the current findings, RRN-VCTE holds promise as a valuable and effective option for patients with RCC and vena cava tumor thrombus, presenting an alternative to traditional open surgeries with potential benefits for the overall well-being and quality of life of patients.
